# High cure rate of periprosthetic hip joint infection with multidisciplinary team approach using standardized two-stage exchange

**DOI:** 10.1186/s13018-019-1122-0

**Published:** 2019-03-13

**Authors:** Doruk Akgün, Michael Müller, Carsten Perka, Tobias Winkler

**Affiliations:** 1 Charité – Universitätsmedizin Berlin, corporate member of Freie Universität Berlin, Humboldt-Universität zu Berlin, and Berlin Institute of Health, Center for Musculoskeletal Surgery, Berlin, Germany; 2 Charite Universitätsmedizin, Augustenburger Platz 1, 13353 Berlin, Germany

**Keywords:** Hip, Outcome, Periprosthetic joint infection, Two-stage exchange

## Abstract

**Background:**

Two-stage exchange arthroplasty is still the preferred treatment choice for chronic PJI. However, the results remain unpredictable. We analyzed the treatment success of patients with an infected hip prosthesis, who were treated according to a standardized algorithm with a multidisciplinary team approach and evaluated with a strict definition of failure.

**Methods:**

In this single-center prospective cohort study, all hip PJI episodes from March 2013 to May 2015 were included. Treatment failure was assessed according to the Delphi-based consensus definition. The Kaplan-Meier survival method was used to estimate the probability of infection-free survival. Patients were dichotomized into two groups depending on the number of previous septic revisions, duration of prosthesis-free interval, positive culture with difficult-to-treat microorganisms, microbiology at explantation, and microbiology at reimplantation.

**Results:**

Eighty-four patients with hip PJI were the subject of this study. The most common isolated microorganisms were coagulase-negative staphylococci (CNS) followed by *Staphylococcus aureus* and *Propionibacterium*. Almost half of the study cohort (46%) had at least one previous septic revision before admission. The Kaplan-Meier estimated infection-free survival after 3 years was 89.3% (95% CI, 80% to 94%) with 30 patients at risk. The mean follow-up was 33.1 months (range, 24–48 months) with successful treatment of PJI. There were no statistical differences in infect eradication rate among the dichotomized groups.

**Conclusions:**

High infect eradication rates were achieved in a challenging cohort using a standardized two-stage exchange supported by a multidisciplinary approach.

## Introduction

Periprosthetic joint infection (PJI) is a serious and challenging complication following total hip arthroplasty (THA). Despite developments in preventative medicine and identification of multiple risk factors, the incidence of PJI is still around 1% following primary THA [1]. With the growing numbers of THA each year [2, 3], the total number of PJI is also rising, with nearly 52,000 registered revisions for hip PJI in the USA performed between January 1, 2009, and December 31, 2013 [2]. Although the best treatment option of PJI is unclear, two-stage exchange arthroplasty is still the preferred treatment choice for chronic PJI [4] associated with high eradication rates around 90% [5–7]. However, results remain unpredictable and some recent studies are showing failure rates of > 20% with a strict definition of success [8–10]. Furthermore, there is still no consensus about the optimal treatment concept in a two-stage exchange arthroplasty. The most controversial aspects are optimal duration of antibiotic therapy, optimal length of prosthesis-free interval, timing of reimplantation, antibiotic-free period and aspiration prior reimplantation, and the role of reimplantation microbiology [8, 11]. The purpose of this study was to report the outcome of our two-stage revision protocol, in which a multi-disciplinary team guides the management of all patients, and all diagnostic and treatment processes are based on a standardized algorithm.

## Methods

### Study design and population

In this single-center prospectively followed cohort study, all hip PJI episodes from 2013 to 2015 were included, which were treated by a standardized comprehensive diagnostic and therapeutic algorithm with two-stage exchange. Native infected joints, joints with missing data, joints with mega prostheses, and patients with a follow-up period less than 24 months were excluded. The study protocol was reviewed and approved by the institutional ethics committee (EA4/040/14).

### Data collection

On admission, age, gender, comorbidities, history of the infected joint, the score of the Charlson comorbidity index (CCI) [12], laboratory values such as serum C-reactive protein (CRP) and blood leukocytes, and presumed route of infection (intraoperative versus hematogenous) were recorded. In addition, the following data were extracted: number of revision surgeries between stages, length of hospital stay, total duration of antimicrobial therapy, serum CRP value at the time of reimplantation, and microbiological and pathological results of revisions and reimplantation.

### Definitions

In this cohort, PJI was diagnosed according to proposed European Bone and Joint Infection Society (EBJIS) criteria [13], since these criteria were used in several outcome studies [14–16]. The definition for successfully treated PJI was based on the Delphi-based international multidisciplinary consensus [17] and was further modified; treatment was considered as successful, if all of the following criteria were fulfilled at the latest follow-up: (i) infection eradication, characterized by a healed wound without fistula, drainage, or pain, and no recurrence of the infection caused by the same organism; (ii) no subsequent surgical intervention for persistent or perioperative infection after reimplantation surgery; (iii) no occurrence of PJI related mortality; and (iv) no long-term (> 6 months) antimicrobial suppression therapy.

Microorganisms such as rifampin-resistant staphylococci, enterococci, ciprofloxacin-resistant gram-negative bacteria, and fungi were defined as difficult-to-treat (DTT) due to the absence of available antibiofilm-active treatment [18].

### Diagnostic algorithm

Each patient underwent a standardized comprehensive diagnostic algorithm. All patients were evaluated by a thorough physical examination with respect to the clinical patient status and soft tissue conditions. Laboratory tests were performed including C-reactive protein (CRP), and plain anteroposterior and lateral radiographs were made. All patients with suspected PJI underwent a diagnostic arthrocentesis. The synovial fluid analysis included total cell count, differential leukocyte count, culture for aerobic and anaerobic organisms, and histological analysis. A synovial fluid leukocyte count of more than 2000/mm^3^ or a finding with more than 70% neutrophils was our cutoff value for the diagnosis of PJI [18]. A positive histopathology was defined as a mean of ≥ 23 granulocytes per ten high-power fields, corresponding to type 2 or type 3 periprosthetic membrane [19]. In case of a dry tap, patients with chronic painful prosthesis underwent a diagnostic surgery to gain at least five periprosthetic tissue samples for microbiological and histopathological analysis or a diagnostic explantation when the suspicion of infection was very high. The cultures were always incubated for 14 days. In case of fever or systemic infection signs, blood cultures for aerobic and anaerobic organisms were obtained and an intensive search for potential primary focus of infection, such as infectious endocarditis, dental (periodontitis, periapical dental abscess, or dental intervention), urogenital, and gastrointestinal source, was done. The explanted prosthesis was sent to sonication to increase the accuracy of microbiologic diagnosis [20].

### Surgical and antimicrobial treatment

The first stage consisted of removal of all implants, as well as infected and necrotic tissue, bone cement, and all other foreign material with a following irrigation and debridement of the surrounding tissues. In most episodes, a cement spacer was not routinely used, unless dead space management or the fixation of a proximal femur fracture was necessary.

Antibiotic treatment was started intravenously (IV) after taking multiple tissue samples during the explantation or in the case of patients presenting with sepsis preoperatively after synovial aspiration. Each patient underwent a standardized antimicrobial therapy, which was based on a previously published concept [18] under the surveillance of our infectious disease specialists [21]. IV treatment was continued for the first 2 weeks after surgery and followed by an oral regimen, if possible. In case of a persistent infection (discharging wound and/or increasing CRP without any other focus and/or local signs of infection), an irrigation and debridement (and spacer exchange, if applicable) of the explanted hip joint was performed. All patients received antibiotics until reimplantation surgery without an antibiotic-free period and diagnostic aspiration. A reimplantation was performed, when the wound was healed, soft tissues were ready for surgery, the general status of the patient was suitable, and there was no clinical sign of a persisting infection. If DTT microorganisms were isolated, a longer prosthesis-free interval (> 6 weeks) was preferred [22]. The reimplantation was used in every patient as another opportunity to perform one more thorough debridement of the surrounding soft tissues and bone prior to placement of the definitive components. During each explantation and reimplantation, at least five periprosthetic tissue samples were collected for microbiological analysis. After reimplantation, antibiotics were administered for 2 weeks via the intravenous route followed by an oral biofilm-active (in non-DTT PJI) or non-biofilm-active antimicrobial treatment (in DTT PJI) for a total treatment duration of at least 12 weeks with a minimum of 6 weeks’ antimicrobial course after reimplantation.

A therapy with biofilm-active antibiotics, such as rifampin or fluoroquinolones, was started only after reimplantation, when all drains were removed, the wound was dry, and the bacterial load was reduced by initial antimicrobial therapy not to cause the emergence of resistance [23, 24]. If medically stable, patients received antimicrobial therapy at home through a peripherally inserted central catheter (PICC) line, when oral antimicrobial therapy was not possible due to multiple drug resistance.

In case of a relevant positive culture during reimplantation (≥ 2 samples were positive for the same microorganism), or a polymicrobial infection (or if the isolated microorganism was the same as the initial infecting organism even if only one culture was positive), antimicrobial therapy was continued for 12 weeks after reimplantation. Otherwise, the standard therapy was given for 6 weeks after reimplantation as planned. A chronic antibiotic suppression was used for patients with increased risk of relapse, including a history of multiple joint infections, deficient immune system, and comorbidities predisposing to PJI [25], after individualized decision-making through a multidisciplinary team, including infectious disease specialists, internal medicine specialists, and orthopedic surgeons, who were involved in every stage of PJI management for each patient.

### Outcome analysis

Patients were seen in the outpatient clinic after 3, 6, and 12 months and after that period annually. Clinical, laboratory, and radiological evaluation were performed by an orthopedic surgeon and an infectious disease specialist.

### Statistical analysis

Patients were dichotomized into two groups depending on the number of previous septic revisions (no previous septic revision vs. ≥ 1 previous septic revision), duration of prosthesis-free interval (short < 6 weeks vs. long > 6 weeks), positive culture with DTT microorganisms (DTT vs. non-DTT), microbiology at explantation (polymicrobial vs. monomicrobial), and microbiology at reimplantation (positive vs. negative). A two-tailed Fisher’s exact test was employed to find significant differences between dichotomized groups. The probability of infection-free survival and the respective 95% confidence interval (95% CI) was estimated using the Kaplan-Meier survival method. Statistical analysis was performed using SPSS version 20 software (SPSS Inc., Chicago, IL) and the software Prism (Version 7.01; GraphPad, La Jolla, CA, USA). A *p* value < 0.05 was considered significant.

## Results

A total of 93 two-stage septic revision hip arthroplasties were performed from 2013 to 2015 at our institution. Three patients died due to non-PJI-related causes. One patient from the failure group died due to myocardial infarction. Two further patients died after 8 and 14 months of follow-up due to an intracerebral hemorrhage and cardiorespiratory failure, respectively. The latter two patients were excluded from further analysis due to short-term follow-up. After applying the exclusion criteria described above, 84 patients with hip PJI were the subject of this study. The presumed route of infection was perioperative in 72 and hematogenous in 12 episodes. Further demographic, clinical, and laboratory characteristics of the cohort are summarized in Table 1. The mean follow-up was 33.1 months (range, 24–48 months) with successful treatment of PJI.

**Table 1:** Patient demographic, clinical, and outcome characteristics

Variable	Hip PJI, *n* = 84
Age, years∗	70 ± 9
CCI (age-adjusted)∗	4 ± 1.9
Previous septic revision^⧫^	39 (46)
1 septic revision	17
2 septic revisions	8
> 2 septic revisions	14
CRP at admission (mg/l)∗	44.9 ± 76.9
Microbiology^⧫^	
Monomicrobial	35 (42)
Polymicrobial	38 (45)
Negative	11 (13)
Difficult-to-treat	18 (21)
Time to reimplantation (day)∗	61 ± 29.8
Short (< 6 weeks)^⧫^	18 (21)
Long (> 6 weeks)^⧫^	66 (79)
Surgery in prosthesis-free interval^⧫^	13 (16)
Total duration of antibiotic therapy (days)∗	116 ± 35.1
Total length of hospital stay (days)∗	33.8 ± 17.5
Positive microbiology at reimplantation^⧫^	18 (21)
CRP at reimplantation (mg/l)∗	13.6 ± 14.9
Treatment failure^⧫^	9 (11.7)

∗The values are given as the mean and the standard deviation

^⧫^The values are given as the number with the percentage of the group in parentheses

We identified microorganisms in 73 of 84 cases (88%). The most common isolated microorganism was coagulase-negative staphylococci (CNS) followed by *Staphylococcus aureus* and *Propionibacterium* (Table 2).

**Table 2:** Microbiology at explantation, reimplantation, and reinfection

	Microorganism	No. (%)
Explantation, *n* = 84	CNS	52 (62)
	*S. aureus*	13 (15)
	*Propionibacterium*	13 (15)
	*Enterococcus* spp.	11 (13)
	*Streptococcus* spp.	5 (6)
	Gram-negative	3 (4)
	Others	13 (15)
Reimplantation, *n* = 18	CNS	14 (78)
	*S. aureus*	2 (11)
	*Propionibacterium*	2 (11)
	Others	2 (11)
	Polymicrobial	3 (17)
Reinfection, *n* = 9	Negative	3 (33)
	*Klebsiella pneumoniae*	2 (22)
	*Escherichia coli*	1 (11)
	*Staphylococcus capitis*	1 (11)
	*Candida* spp.	1 (11)
	*Enterobacter cloacae*	1 (11)

Thirteen patients (16%) underwent at least one revision surgery during the prosthesis-free interval due to persistent infection, which was performed once in eight episodes, twice in one episode, three times in two episodes, four times in one episode, and six times in another episode.

**Fig. 1:**
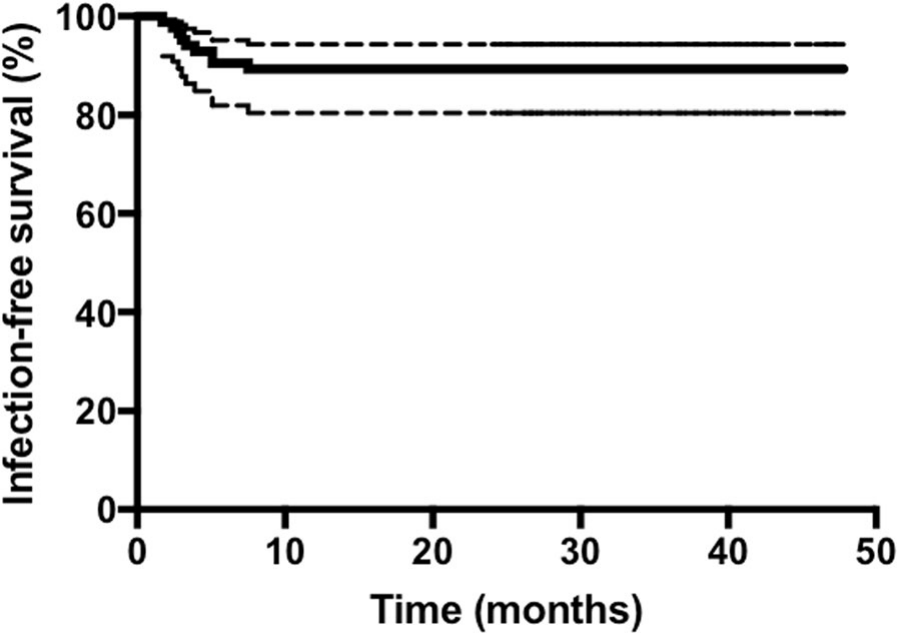
Kaplan-Meier survivorship graph showing the infection-free survival of 84 hip PJI patients. The dotted lines represent the 95% confidence intervals

The mean time interval between stages was 8.7 weeks (range, 1–25 weeks). 21.4% of the patients (18 of 84) underwent a reimplantation after a short interval (< 6 weeks). 21.4% of patients (18 of 84) had a positive culture at the time of reimplantation, and six of these 18 patients (33.3%) with a positive culture at reimplantation underwent a two-stage exchange after a short interval of < 6 weeks. The same microorganism was isolated at reimplantation as the initially isolated microorganism in 7 of these 18 patients (39%). The Kaplan-Meier-estimated infection-free survival after 3 years was 89.3% (95% CI, 80% to 94%) with 30 patients at risk. The survivorship of these patients is illustrated in Fig. 1.

Interestingly, the microorganisms causing reinfection were in none of the nine failures, the same as isolated initially or at the time of reimplantation. The microbiology results of explantation, reimplantation, and reinfection are summarized in Table 2.

Six of 9 failures were early failures within 4 weeks after reimplantation and underwent an irrigation and debridement followed by a 12-week course of antimicrobial treatment. One patient had a reinfection with *Candida* spp. and underwent a two-stage revision with long-term antimicrobial suppression. One patient had a resection arthroplasty and did not get reimplanted due to low-demand and critical health status. Another patient underwent a further two-stage revision, which failed again, so a reimplantation was not performed due to high risk of reinfection.

There were no statistical differences in infect eradication rate among the dichotomized groups (Table 3).

**Table 3:** Dichotomized data for the 84 patients

Dichotomized groups	Numbers	Failure	*p* value
Previous septic revision			0.29
≥ 1	39	6	
0	45	3	
DTT	18	3	0.4
Non-DTT	66	6	
Duration of prosthesis-free interval			0.2
Short (< 6 weeks)	18	0	
Long (> 6 weeks)	66	9	
Microbiology			1.0
Polymicrobial	38	4	
Monomicrobial	35	4	
Microbiology at reimplantation			0.4
Positive	18	3	
Negative	66	6	

## Discussion

Although two-stage exchange arthroplasty is being practiced for more than 20 years in treatment of PJI, results remain unpredictable due to the lack of established standardization, and success rates in the literature are reported to be between 76 and 100% [5–8, 26–29] with varied definition of failure (Table 4). The lack of consensus regarding what constitutes a successful treatment for PJI makes it difficult to compare the results of many studies. Furthermore, the retrospective design and including patients without standardized antimicrobial and surgical treatment algorithm causes inhomogeneous study cohorts, which are difficult to compare [8, 10, 30–32]. Our study was specifically designed to evaluate the infect eradication outcome in an antimicrobially and surgically homogenously treated cohort with a strict definition of treatment failure. In addition, previous septic revision and multiresistant microorganisms were not set as exclusion criteria. Some studies [6, 10, 28] set these characteristics as exclusion criteria, as prior revisions and multiresistancy were reported to be associated with worse outcomes [29, 33]. This was not confirmed using our treatment algorithm. Despite this, our outcome results in a patient cohort where almost every second a patient had a previous septic failure surgery were comparable with the current literature.

**Table 4:** Reported rates of infection eradication in literature with two-stage exchange

Study	Number of patients	Period of study	Definition of failure	Rate of infection eradication (%)
Chen et al. [5]	155 hips	2001–2010	Repeated operation Long-term antibiotics	91.7
Oussedik et al. [7]	39 hips	1999–2002	Recurrent infection	96
Tan et al. [8]	186 knees 81 hips	1999–2013	Delphi-based definition [17]	76
Lange et al. [26]	82 hips	2003–2008	Kamme et al. [47]	85.4
Triantafyllopoulos et al. [27]	239 knees 261 hips	1998–2014	Wound healing problems Elevated ESR/CRP Long-term suppression	91.2
Fink et al. [28]	36 hips	2002–2006	Clinical signs of infection CRP more than 10 mg/dl Osteolysis	100
Berend et al. [30]	186 hips	1996–2009	Further surgery for infection	83
Ibrahim et al. [6]	125 hips	2000–2008	Recurrence of infection	
Leung et al. [48]	50 hips	1998–2006	Recurrence of infection	79

A potential disadvantage of two-stage exchange arthroplasty is the high reported mortality. Ibrahim et al. [6] showed, despite a high rate of infect eradication, a mortality rate of 15% (19 patients), which was also confirmed by Berend et al. [30]. Gomez et al. [10] suggested that the success of two-stage revision arthroplasty be considered from the point of explantation rather than the following reimplantation to account for failures. Lange et al. [26] also showed in his study that only 63% of his study cohort (82 of 130 hips) was reimplanted. Patients reimplanted were younger and had lower CCI and a 68% lower mortality risk in the follow-up period. Unlike the previous studies, we could perform reimplantation in all of our patients, but one (not involved in the cohort of 93 patients), and only three out of 93 patients died of causes unrelated to PJI after reimplantation in our short-term follow-up. It is well known that higher CCI and patients with previous septic revisions with subsequent failure combined with insufficient antimicrobial treatment are associated with a higher risk of reinfection and mortality, so, we propose that medical optimization of these patients through a multidisciplinary team prior two-stage revision plays a crucial role in reducing the mortality and failure rate [34].

Our results showed similar eradication rates in episodes infected by DTT microorganisms compared to the rest of the cohort. With a long interval as proposed by Zimmerli et al. [22], we achieved good eradication rates, despite the unavailability of an antibiofilm-active agent. In our algorithm, we always treat these microorganisms with a long interval. Furthermore, in individual cases, a long-term suppression therapy can also contribute in reducing the risk of a recurrent infection.

Positive culture during reimplantation was evident in 18 cases without any significantly higher risk for subsequent failure compared to the culture-negative group. We were recently able to show in a cohort of patients with hip and knee PJI (same hip patient cohort used also in this study) that positive culture at reimplantation was independently associated with two times the risk of subsequent failure [16]. Possible reasons for lacking significance in this study could be the lower number of patients, significantly higher rate of failure in PJI involving the knee joint, short follow-up, and differences in used statistical methods (multivariate regression analysis vs. Fisher exact test). Tan et al. identified also recently that the risk of treatment failure was significantly higher and reinfection occurred earlier for the cases with a positive culture at reimplantation [8]. Unlike these studies, previous studies could not show any association between positive culture and worse outcomes [11, 35, 36]. But this seems to be due to the small sample size of these studies. Differently, in our study than in other studies, patients with a relevant culture at reimplantation were administered a prolonged antimicrobial treatment, which could have prevented some failures. Also, in a randomized controlled trial, it was shown that the addition of 3 months of oral antibiotics appeared to improve infection-free survival at short-term follow-up [37]. We recommend, therefore, to always implement an antimicrobial treatment after reimplantation, if possible with antibiofilm-active agents, and treating physicians should be aware of possible worse outcomes associated with positive cultures at reimplantation.

Despite suggestions from many authors, to apply a prosthesis free interval of 2–8 weeks followed by an antibiotic holiday of 2 weeks and preoperative aspiration before proceeding to second stage [4, 38, 39], we postulate that a short interval could be as effective in PJI eradication as the long interval in selected patients. The key to success when using short intervals seems to be the availability of an antibiofilm-active agent. None of our 18 episodes treated with a short interval had a recurrence of infection. We do not recommend waiting for CRP in serum to be normalized and an antibiotic-free period with joint aspiration before reimplantation, as cultures from synovial fluid and CRP seem to be uncertain parameters to exclude persistent infection [40–42]. Waiting for CRP to normalize and an antibiotic-free period with joint aspiration prior reimplantation can delay reimplantation unnecessary.

The implantation of a temporary antibiotic-impregnated spacer in the interim period is used worldwide in two-stage exchange arthroplasty, since it enables preservation of the joint space, ensures high local concentrations of antibiotics, and the reimplantation sometimes can be easier due to the absence of scar tissue in the acetabulum and medullary canal [43]. Nevertheless, spacers may act as a foreign body to which microorganisms may adhere, grow, and maintain infection [44, 45]. Several studies identified biofilm formation on the sonicated spacers and reported the association between an infection of the cement spacer and poor clinical outcome and significantly higher failure and reinfection rate after two-stage exchange arthroplasty [44–46]. Furthermore, in a comparison study from Marczak et al., the reinfection rate was similar in patients with and without spacer and five patients from the spacer group underwent spacer exchange due to recurrence of infection [43]. Additionally, several mechanical complications may occur when cement spacers are used, such as spacer fractures, dislocations, and femoral fractures. In our hands, resection arthroplasty has an important role in two-stage exchange arthroplasty with similar success rates in terms of infection control and should be considered especially in cases treated with shorter intervals and when complications related to spacers are expected.

Our study has some limitations. It is not a controlled but an observational study and all cases were from one single center. The mean follow-up time of 2 years is considered to be only a short time [17], and a longer follow-up is needed not to miss a possible relapse after a few years. Another limitation includes the relatively small sample size of our study despite the sample being large for a single center over 3 years, so the missing significance between dichotomized groups could be attributed to this limitation.

## Conclusion

In conclusion, with a standardized therapeutic algorithm using a two-stage exchange arthroplasty, high infect eradication rates were achieved, irrespective of risk factors predictive of failure. A multidisciplinary team should review each patient and compose an individualized treatment plan based on a strict algorithm.

## Abbreviations

CCI: Charlson comorbidity index
CRP: C-reactive protein
DTT: Difficult-to-treat
IV: Intravenously
PJI: Periprosthetic joint infection
THA: Total hip arthroplasty

## References

[1] Engesaeter LB, Dale H, Schrama JC, Hallan G, Lie SA (2011). Surgical procedures in the treatment of 784 infected THAs reported to the Norwegian Arthroplasty Register. Acta Orthop.

[2] Gwam CU, Mistry JB, Mohamed NS, Thomas M, Bigart KC, Mont MA (2017). Current epidemiology of revision total hip arthroplasty in the United States: National Inpatient Sample 2009 to 2013. J Arthroplast.

[3] Tanenbaum JE, Knapik DM, Wera GD, Fitzgerald SJ (2017). National incidence of patient safety indicators in the total hip arthroplasty population. J Arthroplasty.

[4] Cooper HJ, Della Valle CJ (2013). The two-stage standard in revision total hip replacement. Bone Joint J.

[5] Chen SY, Hu CC, Chen CC, Chang YH, Hsieh PH (2015). Two-stage revision arthroplasty for periprosthetic hip infection: mean follow-up of ten years. Biomed Res Int.

[6] Ibrahim MS, Raja S, Khan MA, Haddad FS (2014). A multidisciplinary team approach to two-stage revision for the infected hip replacement: a minimum five-year follow-up study. Bone Joint J..

[7] Oussedik SI, Dodd MB, Haddad FS (2010). Outcomes of revision total hip replacement for infection after grading according to a standard protocol. J Bone Joint Surg Br.

[8] Tan TL, Gomez MM, Manrique J, Parvizi J, Chen AF (2016). Positive culture during reimplantation increases the risk of subsequent failure in two-stage exchange arthroplasty. J Bone Joint Surg Am.

[9] Kheir MM, Tan TL, Gomez MM, Chen AF, Parvizi J (2016). Patients with failed prior two-stage exchange have poor outcomes after further surgical intervention. J Arthroplasty.

[10] Gomez MM, Tan TL, Manrique J, Deirmengian GK, Parvizi J (2015). The fate of spacers in the treatment of periprosthetic joint infection. J Bone Joint Surg Am.

[11] Puhto AP, Puhto TM, Niinimaki TT, Leppilahti JI, Syrjala HP (2014). Two-stage revision for prosthetic joint infection: outcome and role of reimplantation microbiology in 107 cases. J Arthroplast.

[12] Charlson ME, Pompei P, Ales KL, MacKenzie CR (1987). A new method of classifying prognostic comorbidity in longitudinal studies: development and validation. J Chronic Dis.

[13] Ochsner PEBO, Bodler PM, Broger I, Eich G, Hefti F, Maurer T, Nötzli H, Seiler S, Suva D, Trampuz A, Uckay I, Vogt M, Zimmerli W (2016). Infections of the musculoskeletal system. Basic principles, prevention, diagnosis and treatment.

[14] Akgun D, Trampuz A, Perka C, Renz N (2017). High failure rates in treatment of streptococcal periprosthetic joint infection: results from a seven-year retrospective cohort study. Bone Joint J..

[15] Perez-Prieto D, Portillo ME, Puig-Verdie L, Alier A, Martinez S, Sorli L (2017). C-reactive protein may misdiagnose prosthetic joint infections, particularly chronic and low-grade infections. Int Orthop.

[16] Akgun D, Muller M, Perka C, Winkler T (2017). A positive bacterial culture during re-implantation is associated with a poor outcome in two-stage exchange arthroplasty for deep infection. Bone Joint J.

[17] Diaz-Ledezma C, Higuera CA, Parvizi J (2013). Success after treatment of periprosthetic joint infection: a Delphi-based international multidisciplinary consensus. Clin Orthop Relat Res.

[18] Zimmerli W, Trampuz A, Ochsner PE (2004). Prosthetic-joint infections. N Engl J Med.

[19] Krenn V, Morawietz L, Perino G, Kienapfel H, Ascherl R, Hassenpflug GJ (2014). Revised histopathological consensus classification of joint implant related pathology. Pathol Res Pract.

[20] Trampuz A, Piper KE, Jacobson MJ, Hanssen AD, Unni KK, Osmon DR (2007). Sonication of removed hip and knee prostheses for diagnosis of infection. N Engl J Med.

[21] Renz N, Perka C, Trampuz A (2016). Management of periprosthetic infections of the knee. Orthopade..

[22] Zimmerli W, Moser C (2012). Pathogenesis and treatment concepts of orthopaedic biofilm infections. FEMS Immunol Med Microbiol.

[23] Sendi P, Zimmerli W (2012). Antimicrobial treatment concepts for orthopaedic device-related infection. Clin Microbiol Infect.

[24] Nana A, Nelson SB, McLaren A, Chen AF (2016). What’s new in musculoskeletal infection: update on biofilms. J Bone Joint Surg Am.

[25] Osmon DR, Berbari EF, Berendt AR, Lew D, Zimmerli W, Steckelberg JM (2013). Diagnosis and management of prosthetic joint infection: clinical practice guidelines by the Infectious Diseases Society of America. Clin Infect Dis.

[26] Lange J, Troelsen A, Soballe K (2016). Chronic periprosthetic hip joint infection. A retrospective, observational study on the treatment strategy and prognosis in 130 non-selected patients. PLoS One.

[27] Triantafyllopoulos GK, Memtsoudis SG, Zhang W, Ma Y, Sculco TP, Poultsides LA (2016). Periprosthetic infection recurrence after 2-stage exchange arthroplasty: failure or fate?. J arthroplasty.

[28] Fink B, Grossmann A, Fuerst M, Schafer P, Frommelt L (2009). Two-stage cementless revision of infected hip endoprostheses. Clin Orthop Relat Res.

[29] Siqueira MB, Saleh A, Klika AK, O'Rourke C, Schmitt S, Higuera CA (2015). Chronic suppression of periprosthetic joint infections with oral antibiotics increases infection-free survivorship. J Bone Joint Surg Am.

[30] Berend KR, Lombardi AV, Morris MJ, Bergeson AG, Adams JB, Sneller MA (2013). Two-stage treatment of hip periprosthetic joint infection is associated with a high rate of infection control but high mortality. Clin Orthop Relat Res.

[31] McArthur BA, Abdel MP, Taunton MJ, Osmon DR, Hanssen AD (2015). Seronegative infections in hip and knee arthroplasty: periprosthetic infections with normal erythrocyte sedimentation rate and C-reactive protein level. Bone Joint J..

[32] Mittal Y, Fehring TK, Hanssen A, Marculescu C, Odum SM, Osmon D (2007). Two-stage reimplantation for periprosthetic knee infection involving resistant organisms. J Bone Joint Surg Am.

[33] Kilgus DJ, Howe DJ, Strang A (2002). Results of periprosthetic hip and knee infections caused by resistant bacteria. Clin Orthop Relat Res.

[34] Cochran AR, Ong KL, Lau E, Mont MA, Malkani AL (2016). Risk of reinfection after treatment of infected total knee arthroplasty. J Arthroplast.

[35] Bejon P, Berendt A, Atkins BL, Green N, Parry H, Masters S (2010). Two-stage revision for prosthetic joint infection: predictors of outcome and the role of reimplantation microbiology. J Antimicrob Chemother.

[36] Hart WJ, Jones RS (2006). Two-stage revision of infected total knee replacements using articulating cement spacers and short-term antibiotic therapy. J Bone Joint Surg Br..

[37] Frank JM, Kayupov E, Moric M, Segreti J, Hansen E, Hartman C (2016). The Mark Coventry, MD, Award: Oral antibiotics reduce reinfection after two-stage exchange: a multicenter, randomized controlled trial. Clin Orthop Relat Res.

[38] Gehrke T, Alijanipour P, Parvizi J (2015). The management of an infected total knee arthroplasty. Bone Joint J.

[39] Kuzyk PR, Dhotar HS, Sternheim A, Gross AE, Safir O, Backstein D (2014). Two-stage revision arthroplasty for management of chronic periprosthetic hip and knee infection: techniques, controversies, and outcomes. J Am Acad Orthop Surg.

[40] Hoell S, Moeller A, Gosheger G, Hardes J, Dieckmann R, Schulz D (2016). Two-stage revision arthroplasty for periprosthetic joint infections: what is the value of cultures and white cell count in synovial fluid and CRP in serum before second stage reimplantation?. Arch Orthop Trauma Surg.

[41] Janz V, Bartek B, Wassilew GI, Stuhlert M, Perka CF, Winkler T (2016). Validation of synovial aspiration in girdlestone hips for detection of infection persistence in patients undergoing 2-stage revision total hip arthroplasty. J Arthroplast.

[42] Preininger B, Janz V, von Roth P, Trampuz A, Perka CF, Pfitzner T (2017). Inadequacy of joint aspiration for detection of persistent periprosthetic infection during two-stage septic revision knee surgery. Orthopedics.

[43] Marczak D, Synder M, Sibinski M, Polguj M, Dudka J, Kowalczewski J (2017). Two stage revision hip arthroplasty in periprosthetic joint infection. Comparison study: with or without the use of a spacer. Int Orthop.

[44] Sorli L, Puig L, Torres-Claramunt R, Gonzalez A, Alier A, Knobel H (2012). The relationship between microbiology results in the second of a two-stage exchange procedure using cement spacers and the outcome after revision total joint replacement for infection: the use of sonication to aid bacteriological analysis. J Bone Joint Surg Br..

[45] Mariconda M, Ascione T, Balato G, Rotondo R, Smeraglia F, Costa GG (2013). Sonication of antibiotic-loaded cement spacers in a two-stage revision protocol for infected joint arthroplasty. BMC Musculoskelet Disord.

[46] Nelson CL, Jones RB, Wingert NC, Foltzer M, Bowen TR (2014). Sonication of antibiotic spacers predicts failure during two-stage revision for prosthetic knee and hip infections. Clin Orthop Relat Res.

[47] Kamme C, Lindberg L (1981). Aerobic and anaerobic bacteria in deep infections after total hip arthroplasty: differential diagnosis between infectious and non-infectious loosening. Clin Orthop Relat Res.

[48] Leung F, Richards CJ, Garbuz DS, Masri BA, Duncan CP (2011). Two-stage total hip arthroplasty: how often does it control methicillin-resistant infection?. Clin Orthop Relat Res.

